# Calcitonin Gene‐Related Peptide (CGRP)‐Containing Terminals in the Central Amygdala of Mice and Monkeys: Ultrastructural Analysis and Subsynaptic Expression of GluD1

**DOI:** 10.1111/ejn.70607

**Published:** 2026-07-16

**Authors:** Diane Choi, Rosa M. Villalba, Karina Dalal, Jean‐Francois Paré, Shashank M. Dravid, Yoland Smith

**Affiliations:** ^1^ Graduate Program in Molecular and Systems Pharmacology Emory University Atlanta Georgia USA; ^2^ Emory National Biomedical Research Center Emory University Atlanta Georgia USA; ^3^ Department of Psychiatry and Behavioral Sciences Texas A&M University College Station Texas USA; ^4^ Department of Neurology Emory University Atlanta Georgia USA

**Keywords:** CGRP, electron microscopy, immunogold, pain, parabrachial, vGluT2

## Abstract

The right central amygdala (CeA) is involved in the processing of emotional‐affective dimensions of pain. Through the spino‐parabrachio‐amygdaloid pain pathway, the CeA receives nociceptive information from calcitonin gene‐related peptide (CGRP) neurons in the parabrachial nucleus. Recent evidence indicates that the glutamate delta 1 (GluD1) receptor, an atypical ionotropic glutamate receptor that largely functions as a synaptogenic molecule involved in the formation and maintenance of synapses, regulates this projection in mice. Despite its strong cellular expression, little is known about the subsynaptic localization of GluD1, and its potential interaction with CGRP terminals, in CeA neurons. To address this issue and further characterize the ultrastructure and synaptic connectivity of CGRP terminals across species, we used single and double immuno‐electron microscopy techniques in mice and monkeys. For all ultrastructural parameters examined, no species difference was found. In both species, CGRP‐positive (CGRP+) terminals formed symmetric or asymmetric synapses with dendrites, symmetric synapses with soma, and less commonly, asymmetric synapses with spines. Almost 90% of CGRP+ terminals forming asymmetric or symmetric synapses expressed vGluT2 immunoreactivity confirming the glutamatergic and peptidergic nature of this projection. Confocal microscopic analyses confirmed the perisomatic association between CGRP+ and GluD1+ puncta in mice and monkeys. At the ultrastructural level, GluD1 was expressed in the core of symmetric axo‐dendritic and axo‐somatic synapses and perisynaptic to asymmetric synapses formed by CGPR+ terminals. These findings demonstrate that the CGRP+ PB‐CeA projection mediates its effects through a heterogeneous population of terminals that display strong synaptic relationships with GluD1 in rodents and primates.

## Graphical abstract

Proportion of calcitonin gene‐related peptide (CGRP)‐positive terminals expresses synaptic or perisynaptic postsynaptic glutamate delta 1 (GluD1) immunoreactivity at symmetric and asymmetric synapses on soma, dendrite, and spine in the mouse and monkey central nucleus of the amygdala (CeA). The micrographs show confocal and electron microscopic images of CGRP/GluD1 co‐localization at axo‐somatic and axodendric synapses.

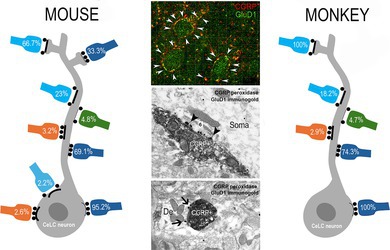

**Abbreviations**
ABC: Avidin biotin peroxidase complex
BSA: Bovine serum albumin
Cbln: Cerebellin
CeA: Central nucleus of the amygdala
CGRP: Calcitonin gene‐related peptide
DCVs: Dense core vesicles
GABA: Gamma aminobutyric acid
GluD1: Glutamate receptor delta 1
KO: Knockout
NDS: Normal donkey serum
PB: Parabrachial nucleus
PBS: Phosphate‐buffered Saline
PKCδ: Protein kinase C‐delta subunit
vGluT2: Vesicular glutamate transporter 2

## 1 Introduction

Approximately 21% of the US adults experience chronic pain (Michaela Rikard et al. 2023) that severely impacts their quality‐of‐life due to physical and emotional burdens. Pain is a complex disorder that consists of multiple dimensions, including sensorimotor, cognitive, and emotional‐affective aspects. The central nucleus of the amygdala (CeA) has been implicated as a key region for the processing of emotional‐affective components of pain (Veinante et al. 2013; Thompson and Neugebauer 2017). In particular, the lateral and capsular divisions of the central amygdala, also coined as the “nociceptive amygdala,” serve as an integrative hub for nociceptive and emotional‐affective processing of pain (Gauriau and Bernard 2002; Thompson and Neugebauer 2017). The CeA receives glutamatergic projections from the external lateral parabrachial nucleus (PB) via the spino‐parabrachio‐amygdaloid pain pathway (Gauriau and Bernard 2002; Spike et al. 2003; Li and Sheets 2020), which is well‐studied for its role in nociceptive pain modulation (Bernard and Besson 1990; Neugebauer 2015; Thompson and Neugebauer 2017). The PB is the sole source of calcitonin gene‐related peptide (CGRP)‐containing inputs to the CeA (Shimada et al. 1985; Kruger et al. 1988; Schwaber et al. 1988; Dobolyi et al. 2005; D'Hanis et al. 2007), a projection that plays a critical role in the transduction of emotional‐affective pain signals through the mediation of nociceptive synaptic transmission and plasticity (Neugebauer et al. 2003; Han and Neugebauer 2004; Han et al. 2005, 2010; Shinohara et al. 2017). The CGRP+ terminals mainly target protein kinase C δ (PKCδ+) neurons, one of two major cell types in the CeA (Li et al. 2013; Kim et al. 2017; Wilson et al. 2019; Adke et al. 2021), considered “pronociceptive” because their activation increases pain‐related responses (Wilson et al. 2019). Thus, it is presumed that the PB‐CeA CGRP+ projection modulates pain through its synaptic signalling with PKCδ+ neurons in the lateral and capsular divisions of CeA (Wilson et al. 2019; Li and Sheets 2020). However, there remains a significant knowledge gap in our understanding of the structural synaptic signalling occurring at these PB‐CeA synapses.

Recent evidence suggests that glutamate delta receptors (GluDs) may regulate transmission of nociceptive pain signals to the CeA (Gandhi et al. 2021). GluDs are unusual members of the ionotropic glutamate receptor superfamily that do not exhibit typical agonist‐induced ionotropic activity (Lomeli et al. 1993; Kristensen et al. 2016). Instead, they function as synaptogenic molecules, forming trans‐synaptic complexes with the presynaptic proteins neurexin and cerebellin (Cbln) to regulate synapse formation and maintenance (Matsuda et al. 2010; Uemura et al. 2010; Elegheert et al. 2016). The role of GluD1 in synaptic transmission has been explored in various regions, including the cerebellum, hippocampus, and striatum (Kusnoor et al. 2010; Konno et al. 2014; Liu et al. 2020; Tao et al. 2018). A recent study has demonstrated that disruption of GluD1 impairs excitatory transmission at PB‐CeA synapses in mice (Gandhi et al. 2021). Furthermore, dysfunction of the trans‐synaptic signalling between GluD1 and Cbln1, which is heavily expressed in PB neurons (Miura et al. 2006; Otsuka et al. 2016), has been implicated in both inflammatory and neuropathic rodent models of pain (Gandhi et al. 2021). Despite recent evidence that GluD1 regulates transmission of CGRP+ terminals at PB‐CeA synapses (Gandhi et al. 2021), our understanding of the underlying substrate of the functional interactions between GluD1 and CGRP+ PB‐CeA synapses remains incomplete. To address this issue, we used single and double immuno‐electron microscopy (EM) approaches to further characterize the ultrastructure, synaptic organization, and transmitter content of CGRP+ terminals and elucidate their subsynaptic relationships with GluD1 in the mouse and monkey CeA. The extension of this analysis to nonhuman primates strengthens the foundation for the translation of knowledge gained through this study to humans.

## 2 Materials and Methods

### 2.1 Animals

For the characterization of CGRP and GluD1 in the CeA, we used tissue from a total of 8 mice (C57Bl/6 J, 1 female, 7 males, 9–16 weeks old), and 7 rhesus monkeys ( *Macaca mulatta* , 3 females, 4 males, 3–11 years old). Five mice brains were received from Dr. Shashank Dravid's laboratory. The remaining 3 mice and all monkeys were from the Emory National Primate Research Center breeding colonies. All animals were deeply anaesthetized with either isoflurane and an overdose of ketamine (100–150 mg/kg, IP) and dormitor (0.1 mg/kg, IP) for mice or pentobarbital (100 mg/kg, IV) for monkeys. Animals were then transcardially perfused with cold oxygenated Ringer's solution followed by a fixative containing 4% paraformaldehyde and 0.1% glutaraldehyde in phosphate buffer (0.1 M, pH 7.4). All animal procedures were in line with the National Institutes of Health guidelines for the use of animals in research and approved by the Creighton University and Emory University Institutional Animal Care and Use Committee Policies and Procedures (IACUC). Following perfusion, brains were collected and postfixed in 4% paraformaldehyde for 24 h. Tissue blocks were cut in 60‐μm‐thick coronal sections with a vibrating microtome. Given well‐documented evidence for pain lateralization to the right CeA (Carrasquillo and Gereau 2008; Ji and Neugebauer 2009; Allen et al. 2023), all brain tissue used in this study was taken from the right hemisphere.

### 2.2 Antibodies

All commercially available antibodies used in these studies are well characterized and recorded in The Antibody Registry. The specificity of the GluD1 antibody has been previously reported (Hoover et al. 2021) and validated by the lack of staining in the striatum of GluD1 KO mice (Liu et al. 2020; Choi et al. 2025).

### 2.3 Light and Confocal Microscopy (LM) Immunohistochemistry

#### 2.3.1 CGRP Immunoperoxidase Localization Studies

Sections of mice and monkey brain tissue containing the central region of the right CeA (rostrocaudal levels: Bregma −1.5 in mice; −8.5 in monkeys) (Paxinos et al. 2000; Paxinos and Franklin 2001) were placed in sodium borohydride (1% in phosphate buffered saline, PBS 0.01 M, pH 7.4) for 20 min and then rinsed five times in PBS. Sections were then placed in a pre‐incubation solution (1% normal goat serum, 1% bovine serum albumin [BSA], 0.3% Triton‐X‐100, and PBS) for 60 min at room temperature (RT). This was followed by incubation in the primary antibody solution (CGRP antibody [Table 1], 1% normal goat serum, 1% BSA, 0.3% Triton‐X‐100, and PBS) for 24 h at RT. After primary antibody incubation, sections were rinsed in PBS and then placed in the secondary antibody solution (goat antiguinea pig biotinylated IgG [1:200 in PBS; Vector laboratories), 1% normal goat serum, 1% BSA, 0.3% Triton‐X‐100, and PBS] for 90 min at RT. Sections were then rinsed with PBS and placed in an avidin‐biotin‐peroxidase complex (ABC; Vector Laboratories) solution for 90 min at RT. Sections were rinsed in PBS and then with tris (hydroxymethyl)aminomethane (TRIS, 0.05 M, pH 7.6) before being incubated in a solution containing 0.01 M imidazole, 0.005% hydrogen peroxide, and 0.025% 3,3′‐diaminobenzidine tetrahydrochloride (DAB; Sigma) in Tris for 10 min at RT. After several rinses in PBS, sections were mounted on gelatin‐coated slides, dehydrated, and cover‐slipped. Sections were digitally scanned by an Aperio ScanScope CS system (Aperio Technologies, Vista, CA) and analyzed using ImageScope software (Aperio Technologies).

**TABLE 1:** List of primary antibodies used in this study.

Antibody—cat. no.	Host	Vendor	RRID	Dilution
CGRP—414 004	Guinea Pig	Synaptic Systems	AB_2737049	1:5000
GluD1—AF1390	Rabbit	Frontiers Inst. Company Ltd	AB_2571757	1:3000
GABA—A2052	Rabbit	Sigma‐Aldrich	AB_477652	1:1000
vGluT2—VGT‐3 (rodent)	Rabbit	MAb Technologies	AB_2315568	1:5000
vGluT2—VGT2–6 (monkey)	Rabbit	MAb Technologies	AB_2315569	1:1000

#### 2.3.2 GluD1 Immunoperoxidase Localization Studies

Sections at the level of CeA were processed with the ABC immunohistochemical method, as described above, using the primary rabbit anti‐GluD1 antibody (Table 1) and the secondary biotinylated goat antirabbit IgG (1:200 in PBS; Vector Laboratories) solutions.

#### 2.3.3 Confocal Microscopy Immunohistochemistry

**FIGURE 1:**
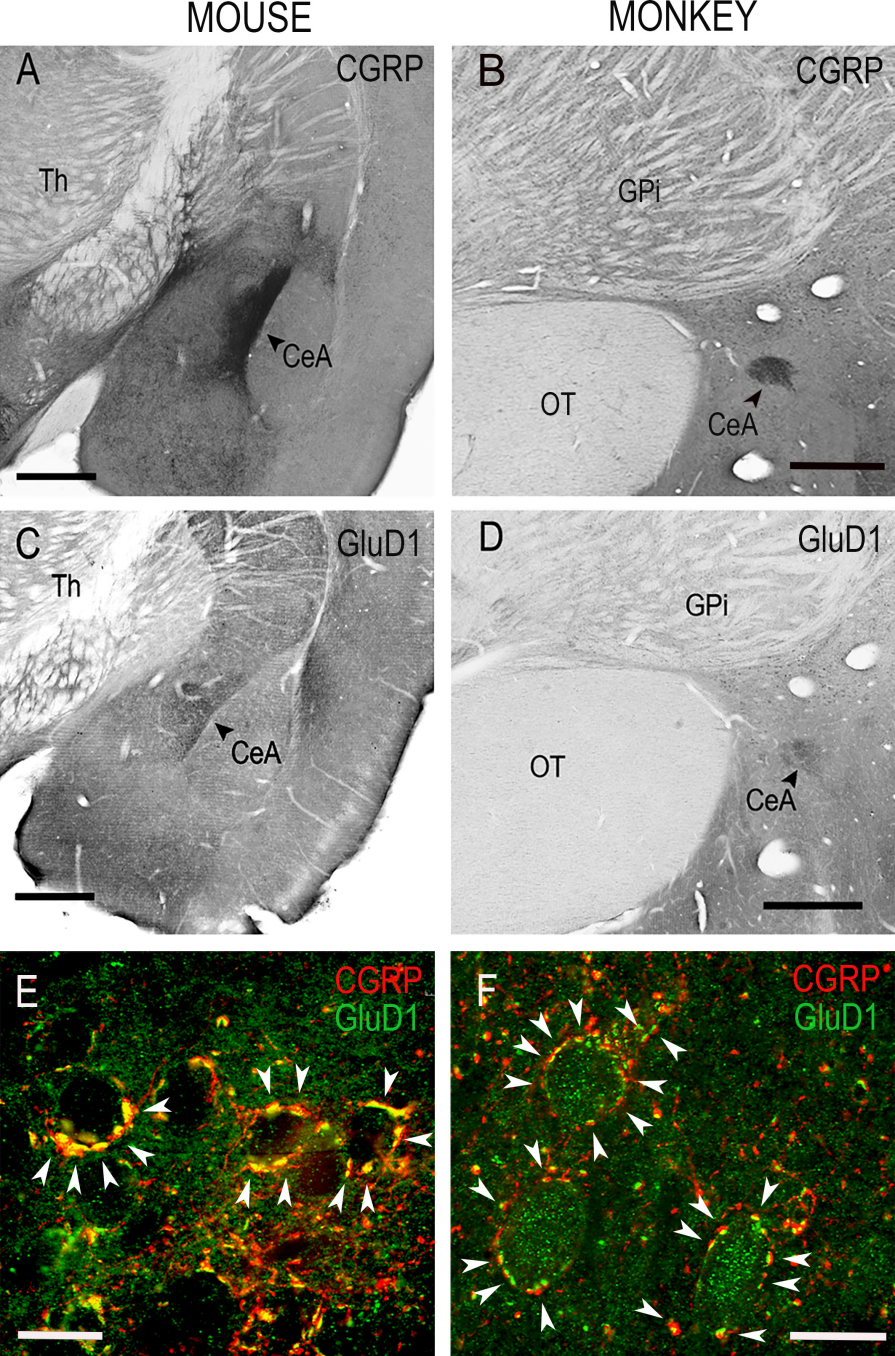
CGRP and GluD1 co‐expression in CeA. (A–D) Light micrographs of CGRP and GluD1 immunoperoxidase staining in a mouse (A, C) and monkey (B, D) right CeA. Note the close overlap between the fields of CGRP and GluD1 labeling in both species. (E, F) Confocal images taken from double‐immunostained tissue in the mouse (E) and monkey (F) CeA. Note the pericellular baskets formed by overlapping CGRP+ (Red) and GluD1 + (Green) puncta around the somata of CeA neurons (white arrowheads). Abbreviations: CeA, central amygdala; GPi, globus pallidus internus; OT, optic tract; Th, thalamus. Scale bar in A, C = 500 μm; B, D = 1 mm; E,F = 20 μm.

To confirm the co‐localization of CGRP+ and GluD1+ puncta into the CeA suggested by the immunoperoxidase data (Figure 1A–D) and a previous mouse study (Gandhi et al. 2021), two sections from the right CeA in 3 mice and 3 monkeys were co‐incubated with both the CGRP and GluD1 antibodies which were respectively localized with rhodamine or FITC‐conjugated secondary antibodies.

In brief, sections were incubated in sodium borohydride (1%) in PBS for 20 min and repeatedly rinsed in PBS. The sections were placed in a preincubation solution containing normal donkey serum (NDS; 5%), BSA (1%), and Triton‐X‐100 (0.3%) for 1 h and then into an incubation solution containing the two specific primary antibodies (GluD1 and CGRP), NDS (1%), and BSA (1%) overnight and then washed in PBS. The sections were then incubated in a solution containing the corresponding secondary antibodies (diluted 1:100) conjugated with rhodamine (CGRP) or FITC (GluD1), NDS (1%), and BSA (1%) for 1 h, rinsed in PBS, mounted with Vectashield (Vector Laboratories) and stored at 4°C. Sections were scanned with a Leica confocal (DM5500B; Leica Microsystems, Bannockburn) and a Hamamatsu camera using Simple PCI software to acquire the images. The analysis for the immunolabelled structures was done using the NIH FIJI software.

### 2.4 EM Immunohistochemistry

#### 2.4.1 Pre‐Embedding CGRP Immunoperoxidase Labeling

After sodium borohydride treatment, sections were placed in a 100% cryoprotectant solution (25% sucrose and 10% glycerol in PB 0.1 M, pH 7.4) for 20 min at RT and then placed in an −80°C freezer for 20 min. Next, sections were reintroduced to 100% cryoprotectant solution for 10 min at RT, followed by diluted (70%, 50%, and 30%) cryoprotectant solutions for 10 min each. Sections then were processed through the same primary and secondary incubation steps as the LM immunohistochemistry method above, with two changes: (1) the omission of Triton‐X‐100 from all solutions and (2) the primary incubation was extended to 48 h at 4°C. After the DAB/peroxidase reaction, sections were rinsed in PB (0.1 M, pH 7.4) and underwent EM processing. In brief, the sections were postfixed in 1% osmium tetroxide solution for 20 min, followed by washes in PB. Sections were then dehydrated with increasing concentrations of alcohol before being placed in propylene oxide. For the 70% alcohol solution, 1% uranyl acetate was added to increase contrast at the EM level. Afterwards, sections were embedded in epoxy resin (Durcupan, ACM; Fluka, Buchs, Switzerland) for 12 h, mounted onto oil‐coated slides, and cover‐slipped before being placed in a 60°C oven for 48 h. Blocks of tissue containing the CeA were taken from the slides, glued on resin blocks, trimmed, and serially cut into 60‐nm sections using an ultramicrotome (Ultra‐cut T2; Leica, Germany Leica). The ultrathin sections were collected onto single slot Pioloform‐coated copper grids, stained with lead citrate for 5 min, and examined with a JEOL electron microscope (JEOL JEM 1011).

#### 2.4.2 Double Pre‐Embedding Immunoperoxidase and Immunogold Labeling

##### 2.4.2.1 CGRP‐GluD1 Co‐Localization

To characterize the relationship between CGRP+ terminals and GluD1 on the surface of CeA neuronal elements, sections from the right CeA were processed for double immuno‐EM localization of CGRP (immunoperoxidase) and GluD1 (pre‐embedding immunogold). After sodium borohydride and cryoprotectant treatments, sections were placed in a pre‐incubation solution containing 5% dry milk and PBS for 30 min at RT. Sections were then rinsed with TBS containing 0.1% gelatin (TBS‐gelatin) and incubated in the primary antibody solution (CGRP primary guinea pig antibody, GluD1 primary rabbit antibody [Table 1], 1% dry milk, and TBS‐gelatin) for 24 h at RT. After rinses in TBS‐gelatin, sections were incubated in the secondary antibody solution (Nanogold‐conjugated goat antirabbit IgG [1:100 in TBS‐gelatin; Nanoprobes Inc.], goat antiguinea pig biotinylated IgG [1:200 in TBS‐gelatin; Vector Laboratories], 1% dry milk, and TBS‐gelatin) for 2 h at RT. Sections were rinsed in TBS‐gelatin and 2% sodium acetate buffer before gold particles were silver intensified with the HQ silver kit (Nanoprobes Inc.) for approximately 16–20 min. After silver intensification, the ABC and DAB procedures were the same as described above in the pre‐embedding immunoperoxidase method. After the DAB reaction, sections were rinsed with PB (0.1 M, pH 7.4) before they underwent EM processing (osmification, dehydration, and embedding) as described in the pre‐embedding immunoperoxidase procedure with the following changes: (1) the sections were fixed in 0.5% osmium tetroxide solution for 10 min instead of 20, and (2) sections were stained with 1% uranyl acetate solution for 10 min instead of 35.

##### 2.4.2.2 CGRP‐vGluT2 Co‐Localization

To assess the extent of the co‐expression of vGluT2 and CGRP in axon terminals, sections from the right CeA were processed for double immuno‐EM localization of CGRP (immunogold) and vGLuT2 (immunoperoxidase) as described above with the following changes: (1) Sections were incubated in the primary antibody solution (CGRP guinea pig primary antibody, vGluT2 primary rabbit antibody) (Table 1), and (2) sections were incubated in the secondary antibody solution (nanogold‐conjugated goat antiguinea pig [1:100 in TBS‐gelatin; Nanoprobes Inc.], goat antirabbit biotinylated IgG [1:200 in TBS‐gelatin; Vector Laboratories]). Because of differences in the COOH terminus between rodents and primates, different well‐characterized vGluT2 primary antibodies were used for rodent and monkey tissues (Table 1).

### 2.5 Data Analysis

All electron micrographs were taken at 40,000X magnification with a CCD camera (Gatan Model 785; Gatan Inc., Pleasanton, CA). Images were analyzed with the Gatan Digital Micrograph software (Version 3.10.1). Some of the micrographs were adjusted for brightness or contrast using either the Digital Micrograph software or Adobe Photoshop (Version 25.9.1). Micrographs were compiled into figures in Adobe Photoshop (Version 25.9.1). Statistical analyses using chi‐squared tests were conducted using R (Version 4.4.1 (2024‐06‐14 ucrt)). Statistical analyses using unpaired *t*‐tests (i.e., comparisons between species) were conducted using GraphPad Prism Software (version 10.4.1 [627]). All graphical data are presented as average percentage values ± standard error of the mean (SEM).

#### 2.5.1 Immunoperoxidase‐Stained Tissue

Data were collected from a total of 6 blocks of right CeA tissue (1 block/animal for 3 mice and 3 monkeys each), with 50–100 electron micrographs taken of randomly selected immunoperoxidase‐labelled CGRP terminals per animal for a total surface area of 2886 μm^2^ in mice and 2190 μm^2^ in monkeys. Labelled terminals were categorized as forming axo‐dendritic, axo‐spinous, or axo‐somatic synapses based on ultrastructural features (Peters et al. 1991). The cross‐sectional diameter of axon terminals was determined by measuring the longest axis of the terminal parallel to the synaptic junction. The relative percentage of CGRP+ terminals forming symmetric versus asymmetric synapses was calculated based on observation of 196 randomly chosen CGRP+ terminals in mice and 160 in monkeys. The mean number of CGRP+ terminals in each category was calculated and statistically compared for each animal species using chi‐squared analyses. The percentage of symmetric (lack of thick postsynaptic density) versus asymmetric (presence of thick postsynaptic density) synapses (Peters et al. 1991) formed by CGRP+ terminals was compared between species using unpaired *t*‐tests.

#### 2.5.2 Double Pre‐Embedding Immunoperoxidase‐ and Immunogold‐Stained Tissue

##### 2.5.2.1 CGRP/vGluT2 Co‐Labeling

To determine the extent of vGluT2 co‐expression in CGRP+ terminals, ultrathin sections of double‐immunostained tissue (CGRP/vGluT2) were examined as described above. Data were collected from a total of 6 blocks of right CeA tissue (1 block/animal for 3 mice and 3 monkeys each). Approximately 60–100 electron micrographs of randomly selected immunogold‐labelled CGRP+ terminals per animal over a total surface area of 3019 μm^2^ in mice and 3197 μm^2^ in monkeys were examined. To avoid false negative results due to differential penetration of antibodies through the tissue sections, all immunogold‐containing CGRP+ terminals were in tissue regions that also contained immunoperoxidase‐labelled vGluT2 + profiles. A minimum of five or more gold particles was required for a terminal to be considered CGRP+. The mean percentage of CGRP+ terminals that co‐expressed vGluT2 immunolabelling was compared between mice (*n* = 275) and monkeys (*n* = 235) using unpaired *t*‐tests.

##### 2.5.2.2 CGRP/GluD1 Co‐Labeling

To determine the synaptic relationships between CGRP+ terminals and postsynaptic GluD1 localization, ultrathin sections of double‐immunostained tissue (CGRP/GluD1) were examined. Data were collected from a total of 6 blocks of right CeA tissue (1 block/animal for 3 mice and 3 monkeys each). Approximately 60–100 electron micrographs of randomly selected CGRP immunoperoxidase‐labelled terminals were taken from the most superficial sections of tissue blocks that contained both reaction products. A minimum of two or more gold particles was required to be considered GluD1‐immunoreactive. A total surface area of 3137 and 2753 μm^2^ of right CeA tissue was examined from mice and monkeys, respectively. The mean percentages of CGRP+ terminals that expressed postsynaptic GluD1 immunogold labeling were calculated in mice (*n* = 197) and monkeys (*n* = 260) and statistically compared for species differences using unpaired *t*‐tests. Postsynaptic GluD1 labeling associated with CGRP+ terminals was defined as “synaptic” when it was found within the core of the synapse or “perisynaptic” when it was located along the edges of the synaptic junctions.

## 3 Results

### 3.1 Light and Confocal Microscopy Data

At the light microscopic (LM) level, strong CGRP neuropil immunoreactivity was found in the mouse and monkey right CeA (Figure 1A,B). The use of adjacent sections immunostained for either CGRP or GluD1 revealed dense overlap in neuropil labeling within the CeA of both species (Figure 1A–D). These observations were confirmed using double immunofluorescence, which showed close registration between CGRP+ and GluD1+ puncta (Figure 1E,F). In line with recent observations (Gandhi et al. 2021), dense pericellular baskets of overlapping CGRP+/GluD1+ puncta were found in both mice and monkeys (Figure 1E,F). These LM observations confirm previous rodent data (Schwaber et al. 1988; Gandhi et al. 2021) and demonstrate that the close relationship between CGRP‐ and GluD1‐containing neuronal elements is also found in the primate CeA.

### 3.2 Ultrastructural Features of CGRP+ Terminals in the CeA

**FIGURE 2:**
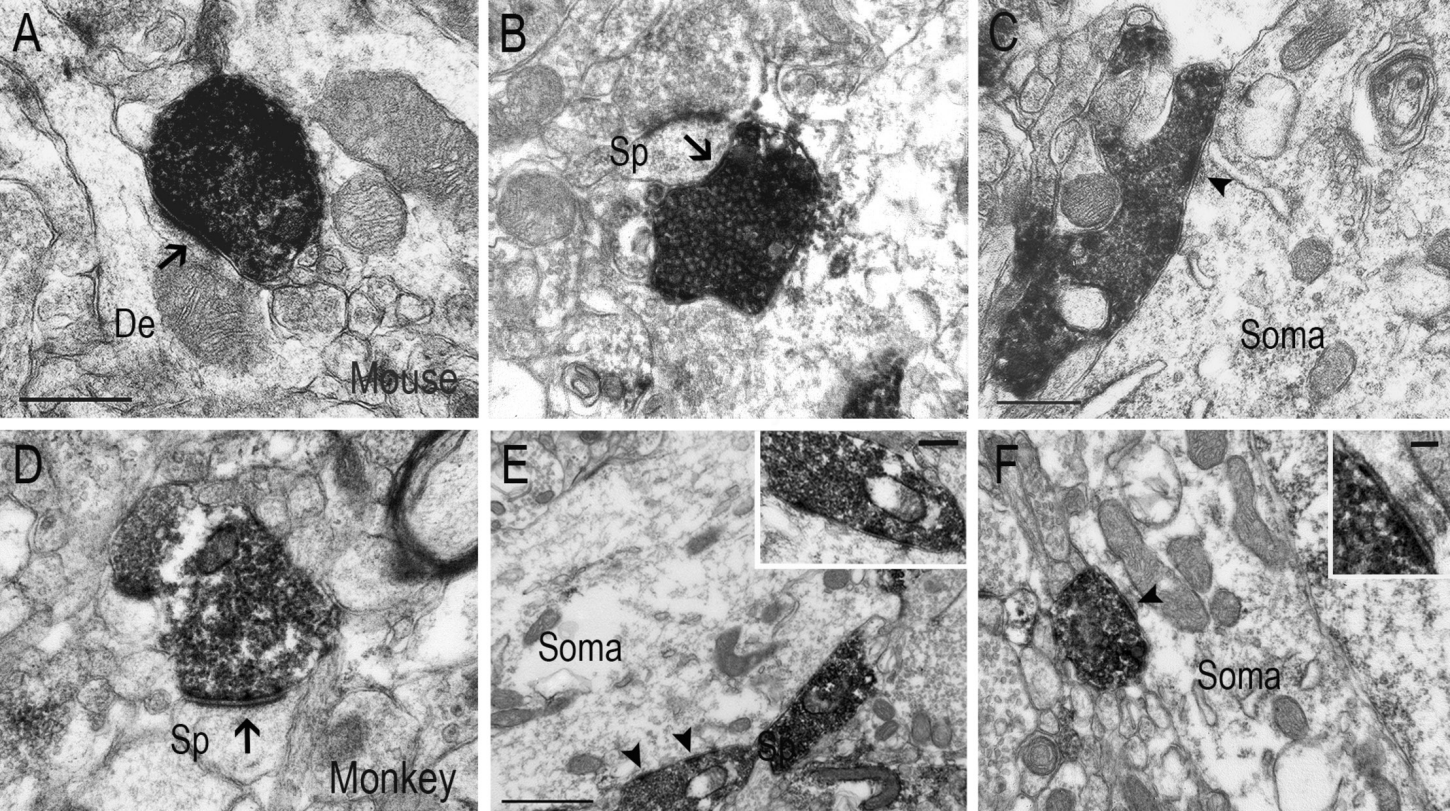
Ultrastructural features of CGRP+ terminals in the mouse and monkey CeA. Examples of CGRP+ terminals in mice (A–C) and monkey (D–F) right CeA. CGRP+ terminals form symmetric (arrowheads) or asymmetric (arrows) axo‐dendritic (A), axo‐spinous (B, D), and axo‐somatic (C, E, F) synapses. Note that many CGRP+ terminals often converge around the soma of CeA neurons (E). Abbreviations: De, dendrite; Sp, spine. Scale bar in A (applies to B) = 0.5 μm. Scale bar in C (applies to D) = 0.5 μm. Scale bar in E (applies to F) = 0.5 μm.

To further characterize and assess potential species differences in the ultrastructural features and synaptic connectivity of CGRP+ terminals within the CeA, CGRP‐immunostained sections from both mice and monkey CeA were analyzed in the transmission electron microscope. In line with our immunofluorescence data (Figure 1E,F), the somata of CeA neurons were often contacted by multiple CGRP+ terminals (Figure 2C,E,F). Most CGRP+ terminals varied in size, ranging from ~0.25 μm to over 1 μm in cross‐sectional diameter, contained numerous small, spherical or oval, electro‐lucent vesicles as well as large dense core vesicles (DCVs) which were occasionally difficult to visualize because of the dense peroxidase deposit (Figure 2). However, in terminals with less intense labeling, numerous large DCVs could be seen (Figure 5D). In tissue from which CGRP was localized with the pre‐embedding immunogold method, the gold particles labeling was commonly found around the external membrane of DCVs confirming their content in CGRP (Figure 4E,F). When they could be visualized, DCVs were distributed throughout the terminals without any specific association with the synaptic active zones (Figures 4E,F and 5D). There were no notable differences in the morphology of CGRP+ terminals between mice and monkeys (Figures 2, 4, and 5).

#### 3.2.1 Postsynaptic Targets of CGRP+ Terminals in the CeA

**FIGURE 3:**
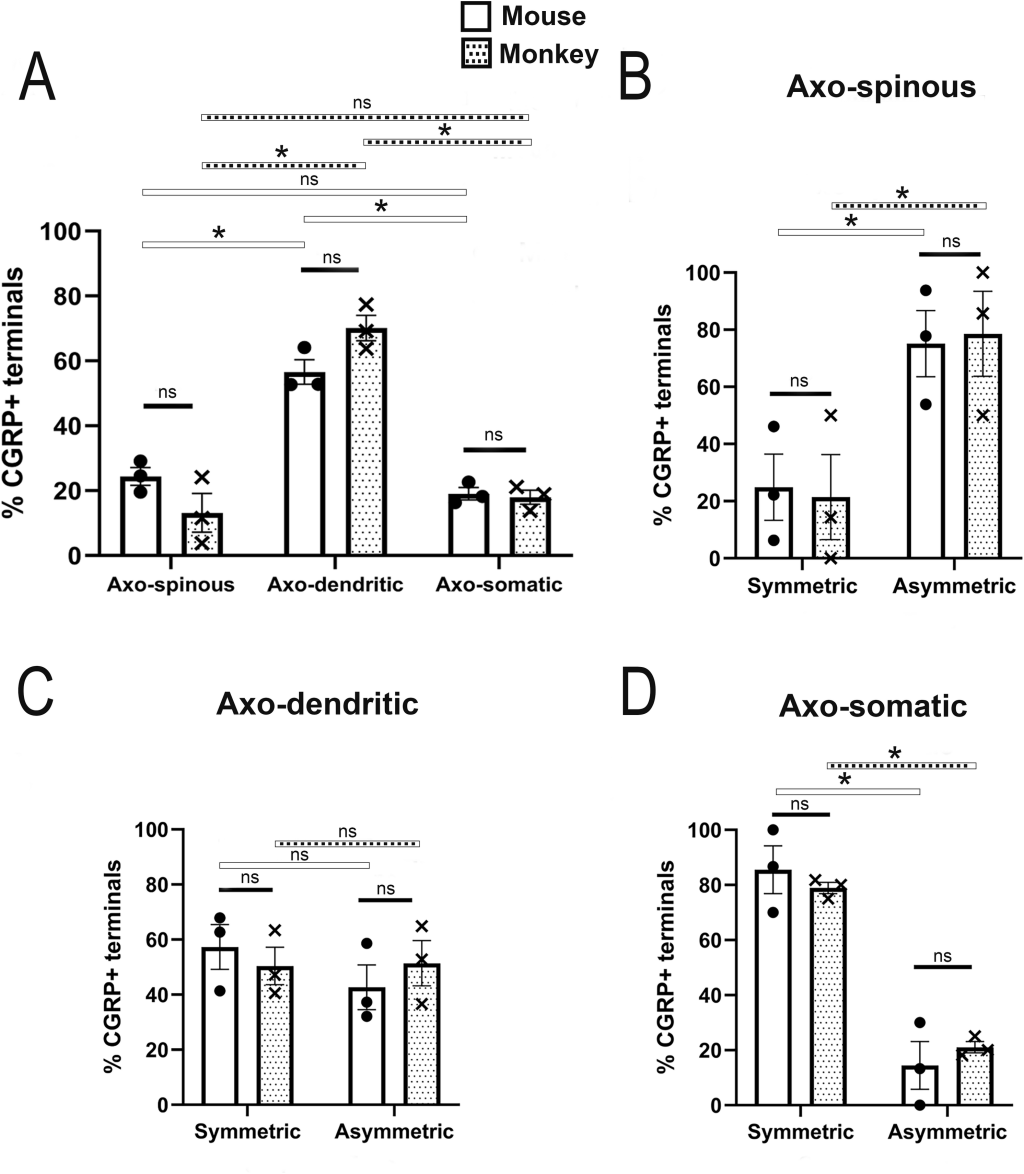
Quantitative analysis of the synaptic connectivity of GGRP+ terminals in mouse and monkey CeA. (A) Bar graph showing the mean percentages (± SEM) of CGRP+ terminals that form axo‐spinous, axo‐dendritic, and axo‐somatic synapses from 3 mice and 3 monkeys. The symbols over each bar indicate the mean values for each animal. CGRP+ terminals form significantly more axo‐dendritic than axo‐spinous and axo‐somatic synapses in both mice and monkeys (chi‐squared test, *p* < 0.001). The relative percentage of synapses formed between CGRP+ terminals and different postsynaptic targets (spines, dendrites, and somata) was conserved across species (unpaired *t*‐test, axo‐spinous, *p* = 0.161; axo‐dendritic, *p* = 0.068; axo‐somatic, *p* = 0.721). (B–D) Graphs showing the mean percentages (± SEM; *N* = 3 mice, 3 monkeys) of CGRP+ terminals forming symmetric or asymmetric axo‐spinous (B), axo‐dendritic (C), or axo‐somatic (D) synapses. (B) Most axo‐spinous synapses were asymmetric, whereas axo‐somatic synapses were symmetric (chi‐squared test, *p* < 0.001). (C) The percentage of axo‐dendritic contacts were evenly distributed between symmetric and asymmetric synapses. (C) The relative percentages of CGRP+ terminals involved in different categories of synapses with each postsynaptic target was conserved across species (unpaired *t*‐test; axo‐spinous, symmetric: *p* = 0.864, asymmetric: *p* = 0.864; axo‐dendritic, symmetric: *p* = 0.548, asymmetric: *p* = 0.491; axo‐somatic, symmetric: *p* = 0.499, asymmetric: *p* = 0.499).

To determine the postsynaptic targets of CGRP+ terminals in the CeA, blocks of immunoperoxidase‐stained tissue from 3 mice and 3 monkeys were examined in the electron microscope (EM). We observed strong CGRP immunoreactivity in presynaptic terminals that formed axo‐dendritic (Figures 2A), axo‐spinous (Figures 2B,D), or axo‐somatic synapses (Figures 2C,E,F). Analysis of approximately 50 micrographs of randomly selected CGRP+ terminals/animal showed that most CGRP+ terminals formed axo‐dendritic synapses in both mice (chi‐squared test, *p* < 0.001) and monkeys (chi‐squared test, *p* < 0.001) (Figure 3A). In mice, 56.6% ± 3.8% of CGRP‐labelled terminals formed axo‐dendritic synapses, while 70.1% ± 3.9% did so in monkeys (Figure 3A). Albeit to a lesser extent, CGRP+ terminals also formed axo‐spinous (24.4% ± 2.7% in mice and 13.2% ± 5.9% in monkeys) and axo‐somatic (19.0% ± 1.9% in mice and 17.9% ± 2.2% in monkeys) synapses (Figure 3A). The distribution of postsynaptic targets to CGRP+ terminals was not significantly different between species (Figure 2; unpaired *t*‐test, axo‐dendritic, *p* = 0.068; axo‐spinous, *p* = 0.161; axo‐somatic, *p* = 0.721).

#### 3.2.2 CGRP+ Terminals Form Symmetric or Asymmetric Synapses in the CeA

Synapses formed by CGRP+ terminals in the CeA were categorized as symmetric or asymmetric based on the absence or presence of a dense postsynaptic density, respectively (Peters et al. 1991). The analysis of 50–100 CGRP+ terminals/animal forming clear synaptic junctions revealed that 55.0% ± 9.8% and 50.9% ± 8.2% formed symmetric synapses (black arrowhead, Figure 2C,E,F), while 45.0% ± 9.8% and 49.1% ± 8.2% established asymmetric synapses (black arrows, Figure 2A,B,D) in mice and monkeys, respectively. However, the prevalence of either type of synapses varied significantly between postsynaptic targets (Figure 3B–D). For instance, most axo‐spinous synapses formed by CGRP+ terminals were asymmetric (75.1% ± 11.6% and 78.6% ± 14.5% in mice and monkeys, respectively) (Figure 3B), whereas most axo‐somatic synapses were symmetric (85.6% ± 8.7% and 78.9% ± 2.0% in mice and monkeys, respectively) (Figure 3D). Axo‐dendritic synapses were divided evenly between asymmetric (42.7% ± 8.1% and 51.4% ± 8.2% in mice and monkeys, respectively) and symmetric (57.3% ± 8.1% and 50.4% ± 6.8% in mice and monkeys, respectively) (Figure 3C) synapses. There was no significant difference in the overall percentage and prevalence of symmetric versus asymmetric synapses on specific postsynaptic targets formed by CGRP+ terminals in the CeA between mice and monkeys (unpaired *t*‐test; axo‐dendritic: symmetric *p* = 0.548, asymmetric *p* = 0.491; axo‐somatic: symmetric *p* = 0.499, asymmetric *p* = 0.499; axo‐spinous: symmetric *p* = 0.864, asymmetric *p* = 0.864).

#### 3.2.3 CGRP+ Terminals Largely Express vGluT2 in the CeA

Although there is strong literature evidence that the PB‐CeA projection is excitatory and glutamatergic (Neugebauer and Li 2002; Sugimura et al. 2016), the fact that a large proportion of CGRP+ terminals in the CeA form symmetric synapses (Figure 3), a type of synaptic junction commonly associated with inhibitory transmission, prompted us to confirm that CGRP+ terminals in the mouse and monkey CeA express vGluT2, the primary glutamatergic marker found in PB neurons (Lein et al. 2007). To determine the co‐expression of CGRP and vGluT2 immunoreactivity in presynaptic terminals in the CeA, blocks of double‐immunostained (CGRP‐vGluT2) tissue from 3 mice and 3 monkeys were examined. In these experiments, CGRP was labelled with immunogold, while immunoperoxidase was used to label vGluT2.

**FIGURE 4:**
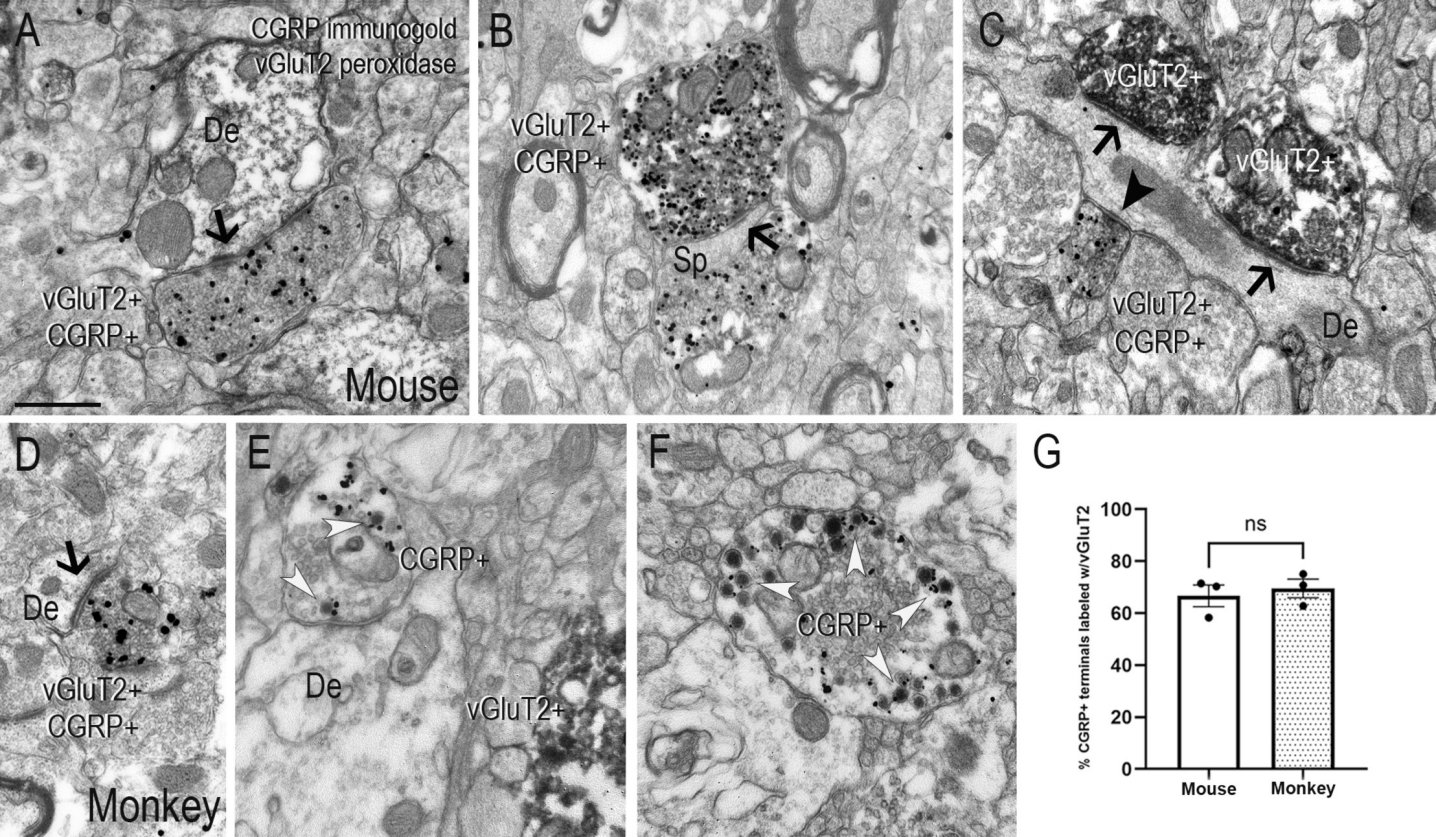
CGRP+ terminals express vGluT2 in the CeA. Micrographs of double‐immunostained terminals for CGRP (immunogold) and vGluT2 (peroxidase) in the mouse (A–C) and monkey (D–F) right CeA. Most CGRP+ terminals forming asymmetric (arrows) or symmetric (arrowheads) synapses co‐express vGluT2 immunoreactivity (A–D). Approximately one third of CGRP+ terminals were devoid of vGluT2 labeling (E, F). In these terminals, the gold labeling was often closely opposed to the external membrane of dense‐core vesicles (white arrowheads in E, F). (G) Bar graph illustrates the mean percentages (± SEM; *N* = 3 mice, 3 monkeys) of CGRP+ terminals labelled with vGluT2. Of all CGRP‐labelled terminals examined (274 in mice, 235 in monkeys), 66.7% ± 4.2% and 69.5% ± 3.6% expressed vGluT2 immunoreactivity in the mouse and monkey CeA, respectively. There were no significant differences in the percentages of double labelled terminals between mouse and monkey (unpaired *t*‐test, *p* = 0.560). Abbreviations: De, dendrite; Sp, spine. Scale bar in A (applies to B–F) = 0.5 μm.

Electron micrographs were only collected from superficial ultrathin sections where both CGRP and vGluT2 labeling were co‐expressed. A threshold of five or more gold particles was required for a terminal to be considered CGRP‐immunoreactive. As expected, we observed frequent co‐expression of vGluT2 and CGRP labeling in both the mouse (Figure 4A–C) and monkey (Figure 4D) CeA. However, there were also CGRP+ terminals that were devoid of vGluT2 labeling (Figure 4E,F). In general, these CGRP+/vGluT2‐ terminals were enriched in DCVs that expressed gold labeling on their external membrane, indicating their content in CGRP (Figure 4E,F). Because strong vGluT2 labeling was observed in other terminals in the close vicinity of the CGRP+/vGluT2‐ terminals (Figure 4E), the lack of vGluT2 immunoreactivity in these CGRP+ terminals is unlikely to be the result of poor antibody penetration issues.

Of all CGRP+ terminals examined (*n* = 274 in mice and *n* = 235 in monkeys), 66.7% ± 4.2% and 69.5% ± 3.6% expressed vGluT2 immunoreactivity in the mouse and monkey CeA, respectively (Figure 4G). There were no significant differences in vGluT2 expression within CGRP+ terminals between the mouse and monkey CeA (Figure 4G; unpaired *t*‐test, *p* = 0.633). Of all CGRP+ terminals that formed clear synaptic junctions (*n* = 63 in mice and *n* = 80 in monkeys), we determined the relative proportion of CGRP+ terminals forming asymmetric and symmetric synapses. As expected, most CGRP+ terminals that formed asymmetric synapses (*n* = 35 in mice and *n* = 37 in monkeys) displayed vGluT2 immunoreactivity (black arrows, Figure 4A,B,D). Similarly, of the CGRP+ terminals involved in symmetric axo‐dendritic or axo‐somatic synapses (*n* = 28 in mice and *n* = 43 in monkeys), 88.9% ± 5.6% and 79.0% ± 1.0% of CGRP+ terminals also displayed vGluT2 immunostaining (Figure 4C), while 11.1% ± 5.6% and 21.0% ± 1.0% were devoid of vGluT2 labeling (Figure 4E,F) in mice and monkeys, respectively. These findings suggest that, in both mice and monkeys, the bulk of CGRP+ PB‐CeA terminals use glutamate as transmitter irrespective of the type of synaptic specializations they are involved in and that a small proportion is devoid of vGluT2 immunoreactivity.

### 3.3 GluD1 Expression at Synapses Formed by CGRP+ Terminals in the CeA

To determine the association between CGRP+ terminals and postsynaptic GluD1 localization, blocks of double‐immunostained (CGRP‐GluD1) tissue from 3 mice and 3 monkeys were examined. In these experiments, CGRP was labelled with immunoperoxidase and GluD1 with immunogold. EM observations were collected from only the most superficial tissue sections where both CGRP and GluD1 labeling were co‐expressed. A threshold of two or more gold particles was required to be considered GluD1‐immunoreactive.

**FIGURE 5:**
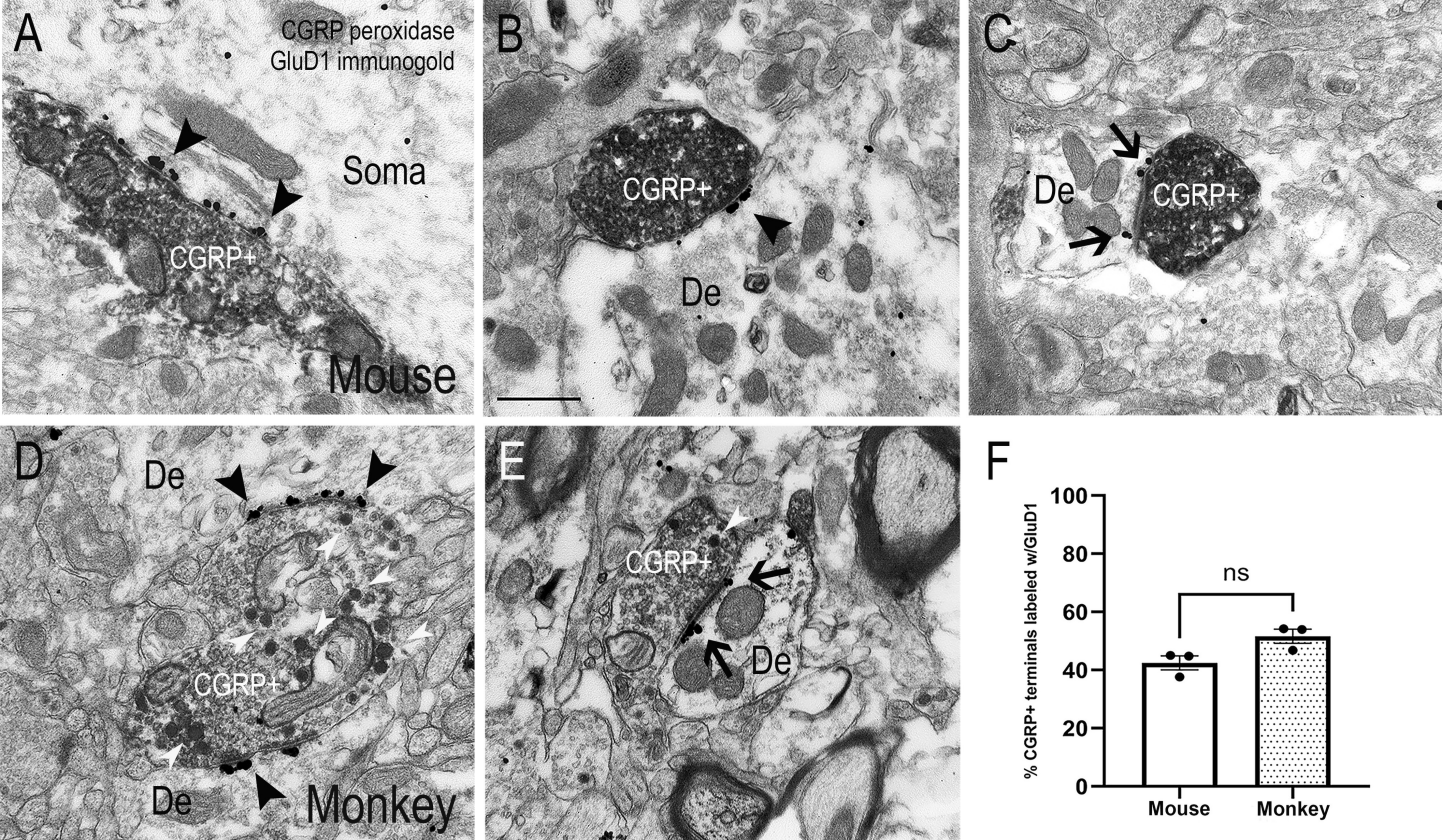
GluD1 is associated with CGRP+ terminals in the CeA. Micrographs of double‐immunostained tissue for GluD1 (immunogold) and CGRP (peroxidase) in the mouse (A–C) and monkey (D–E) right CeA. (A) Synaptic GluD1 labeling (arrowheads) at symmetric axo‐somatic (A) and axo‐dendritic (B, D) synapses. (C, E) Perisynaptic GluD1 labeling (arrows) at asymmetric axo‐dendritic synapses. (F) Mean percentages (± SEM) of synapses formed by CGRP+ terminals that displayed postsynaptic GluD1 labeling in 3 mice and 3 monkeys. No significant species difference was found (unpaired *t*‐test, *p* = 0.056). Abbreviations: De, dendrite. Scale bar in A (applies B–E) = 0.5 μm.

We observed strong GluD1 synaptic labeling within the core of symmetric axo‐dendritic (Figure 5B,D) and axo‐somatic synapses (Figure 5A) formed by CGRP+ terminals, while perisynaptic GluD1 labeling was found at the edges of asymmetric axo‐dendritic and axo‐spinous synapses (Figure 5C,E). Occasionally, multiple CGRP+ terminals associated with postsynaptic GluD1 labeling converged around single somata, confirming the overlap between CGRP+ and GluD1+ puncta seen at the confocal microscopic level (Figure 1E,F) in this and previous study (Gandhi et al. 2021). Of all synapses formed by CGRP+ terminals examined in this material, 42.5% ± 2.4% and 51.6% ± 2.4% expressed GluD1 immunoreactivity in the mouse and monkey CeA, respectively (Figure 5F). Statistical analyses revealed no significant difference in GluD1 expression at CGRP‐labelled terminals between the mouse and monkey CeA (Figure 5F; unpaired *t*‐test, *p* = 0.056).

**FIGURE 6:**
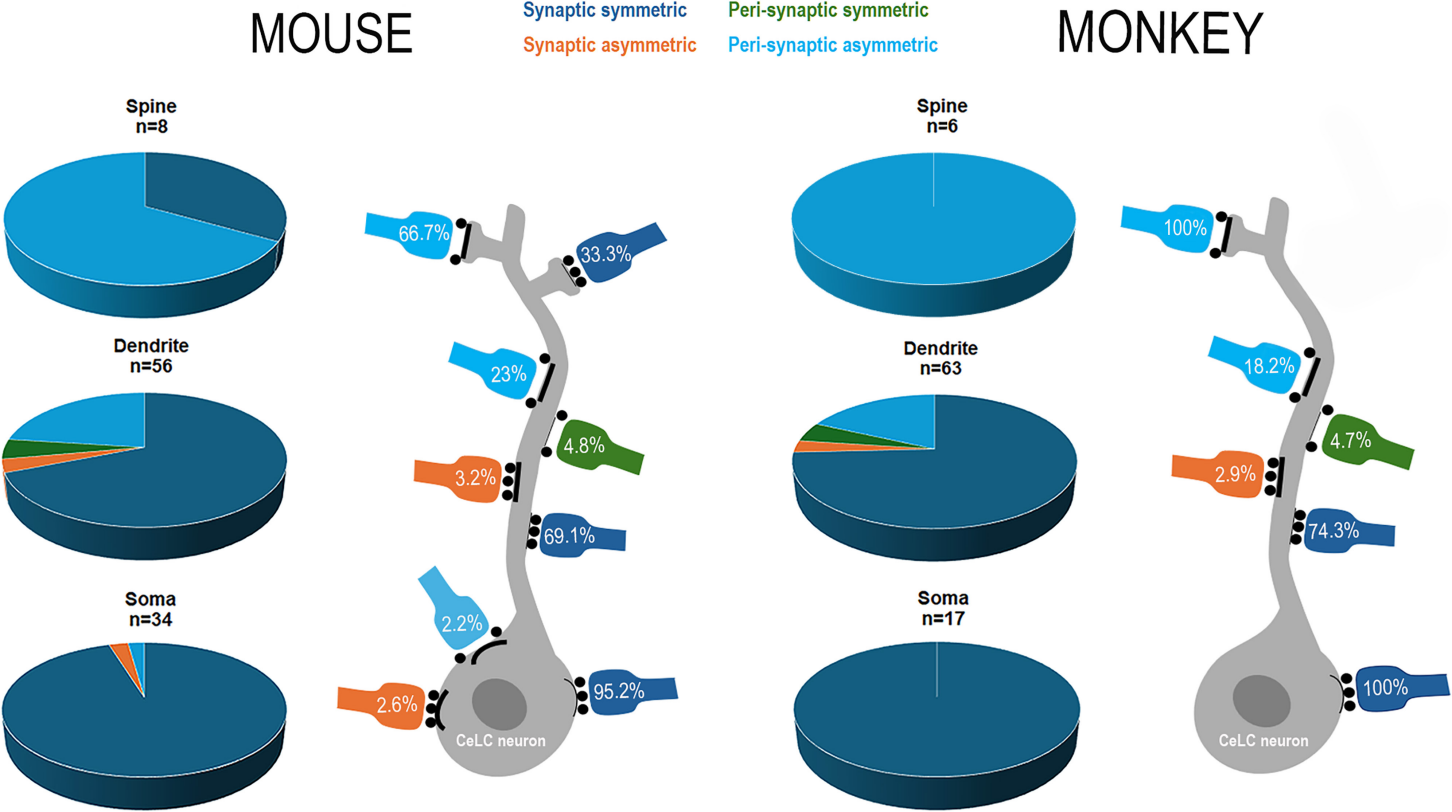
Relative abundance and distribution of synapses formed by CGRP+ terminals in the mouse and monkey CeA. The pie charts represent the percentages of synaptically or perisynaptically GluD1‐labelled symmetric or asymmetric synapses formed by CGRP+ terminals on the soma, dendrites and spines of CeA neurons in mice (left panel) and monkeys (right panel). The “*n*” under each postsynaptic target is the total number of GluD1+/CGRP+ synapses examined. The raw and mean (+/− SEM) values used in these pie charts are shown in Tables S1 and S2. The neuronal schematics depict the relative proportions of the different types of GluD1+/CGRP+ synapses on each neuronal compartment (soma, dendrite, and spine). Both the terminal cartoons and pie charts are color coded with their synaptic subtypes at the top of the figure. The percentage within each terminal schematic represents the relative proportion of the different types of synapses in contact with each neuronal compartment (soma, dendrite, and spine).

Figure 6 shows pie charts that illustrate the relative distribution of GluD1 labeling at symmetric or asymmetric axo‐somatic, axo‐dendritic, or axo‐spinous synapses formed by CGRP+ terminals in the mouse and monkey CeA. Graphical distributions of the pattern of GluD1 expression at synapses formed by CGRP+ terminals on the soma, dendrites, and spines of CeA neurons are also depicted. These charts and summary illustrations were based on data collected from a total of 98 CGRP+ terminals in mice and 88 in monkeys. The mean (+/− SEM) and individual percentages of different types of GluD1 + synapses formed by CGRP+ terminals on the soma, dendrites, and spines of CeA neurons in the 3 mice and 3 monkeys used in this series of experiments are provided in Tables S1 and S2. Overall, the most common type of GluD1+ synapses formed by CGRP+ terminals across the somato‐dendritic domain of CeA neurons were symmetric and exhibited GluD1 labeling in the core of their synaptic junctions (Figure 6). Perisynaptic GluD1 labeling at asymmetric axo‐dendritic and axospinous synapses formed by CGRP+ terminals was also frequently found (Figure 6).

## 4 Discussion

The CeA is a key region for the integration of nociceptive and emotional‐affective processing of pain (Gauriau and Bernard 2002; Veinante et al. 2013; Neugebauer 2015; Neugebauer et al. 2004; Thompson and Neugebauer 2017). Through the spino‐parabrachio‐amygdaloid pain pathway, the CeA receives direct monosynaptic inputs from CGRP‐containing PB neurons (Schwaber et al. 1988; Neugebauer and Li 2002; Sugimura et al. 2016; Li and Sheets 2020). Pain‐related neuroplasticity, such as enhanced transmission and CeA neuronal excitability, at PB‐CeA synapses has been well‐studied in acute inflammatory (Neugebauer et al. 2003; Han et al. 2005; Ren and Neugebauer 2010) and neuropathic (Han and Neugebauer 2004; Ikeda et al. 2007) pain models. However, our knowledge of the anatomical substrate through which nociceptive signals are transmitted through PB‐CeA synapses remains limited.

In the present study, we used immuno‐EM techniques to characterize the ultrastructural features and the pattern of synaptic connections of CGRP+ terminals in the mouse and monkey right CeA. We observed dense CGRP immunoreactivity throughout the CeA neuropil. At the electron microscopic level, CGRP+ terminals formed symmetric or asymmetric synapses with dendritic profiles and asymmetric synapses with spines. In line with previous literature (Shimada et al. 1989), numerous large CGRP+ boutons also formed symmetric synapses with cell bodies. Using double immuno‐EM approaches, we showed that most CGRP+ terminals forming asymmetric axo‐spinous and axo‐dendritic synapses expressed vGluT2, confirming the glutamatergic nature of this projection (Sugimura et al. 2016; Neugebauer and Li 2002). We also found a large proportion of vGluT2+/CGRP+ terminals forming symmetric synapses, while approximately 10% and 20% of CGRP+ terminals forming symmetric synapses were devoid of vGluT2 immunoreactivity in mice and monkeys, respectively.

Given recent data showing that GluD1 regulates excitatory transmission at PB‐CeA synapses and contributes to pain‐related behaviors (Gandhi et al. 2021), we further characterized GluD1 expression at synapses formed by CGRP+ terminals in the CeA nucleus using double immuno‐EM methods. In both mice and monkey CeA tissue, we observed strong postsynaptic GluD1 expression at synapses formed by CGRP+ terminals. Most GluD1 immunogold labeling was found in the core of symmetric synapses or was perisynaptically localized at the edges of asymmetric CGRP+ synapses (Figure 6). Overall, these findings extend recent data showing the importance of GluD1 in regulating PB‐CeA CGRP+ synapses and further demonstrate that CGRP+ terminals in the CeA are heterogeneous in their ultrastructural and chemical phenotypes, raising the possibility that the PB‐CeA projection originates from different subsets of PB neurons. Our results also demonstrate that the chemical and ultrastructural features of PB‐CeA synapses are maintained between rodents and monkeys, providing a strong foundation to translate animals' preclinical findings gathered about this nociceptive pathway to the human brain.

### 4.1 Differential Synaptic Ultrastructure of CGRP+ Synapses: Potential Functional Significance

Given evidence that the right CeA is the key modulator of pain compared to the left CeA in rodents and humans (Carrasquillo and Gereau 2008; Ji and Neugebauer 2009; Allen et al. 2023), all our ultrastructural data have been collected from the right hemisphere. Although pain lateralization has long been recognized, the molecular substrate of this phenomenon remains poorly understood. Whether some of the synaptic features of the right CeA presented in this study also applies to the left side remains to be established. Future studies assessing the effects of GluD1 dysregulation on transmission of PB pain signals through the left and right PB‐CeA circuit combined with postmortem ultrastructural analyses of changes in the morphology and pattern of synaptic connectivity of CGRP+ terminals in both nuclei are warranted to further address this issue.

Our ultrastructural data revealed a heterogeneous population of CGRP+ terminals in the mouse and monkey CeA. Most CGRP+ terminals forming axo‐spinous synapses were asymmetric, while a majority of axo‐somatic synapses were symmetric. CGRP+ terminals forming axo‐dendritic synapses were evenly distributed between asymmetric and symmetric junctions. Given electrophysiological evidence that the PB input to the CeA is glutamatergic and excitatory (Neugebauer and Li 2002; Sugimura et al. 2016), one would expect the majority of CGRP+ terminals to form asymmetric synapses, a type of synapses most commonly associated with glutamatergic excitatory transmission (Peters et al. 1991; Kennedy 1997; Hassel and Dingledine 2012). In agreement with our findings, previous rodent studies have also reported the appearance of PB‐CGRP terminals forming symmetric synapses onto soma and dendritic shafts in the CeA (Shimada et al. 1989; Honkaniemi et al. 1990; Dong et al. 2010; Lu et al. 2015) and the bed nucleus of the stria terminalis (Shimada et al. 1989; Kozicz and Arimura 2001). Our double immuno‐EM data extend these observations showing that the majority of CGRP+ terminals forming symmetric or asymmetric synapses exhibit vGluT2 immunoreactivity, thereby confirming the glutamatergic nature of this projection (Pauli et al. 2022). To our knowledge, there is limited evidence from the literature of vGluT2‐containing terminals forming symmetric synapses. In most brain regions, including the amygdala, these glutamatergic boutons form conventional asymmetric synapses with thick postsynaptic densities (Raju et al. 2008; McDonald et al. 2019; Villalba et al. 2021). Preliminary unpublished data from our laboratory suggest that a subset of terminals from the internal globus pallidus that co‐express vGluT2 and GABA in the monkey lateral habenula occasionally form symmetric synapses (unpublished observations). Given that our EM data were collected from single ultrathin sections, we cannot rule out that some of the synapses categorized as “symmetric” in our study may display a thicker postsynaptic density if followed through serial sections. However, the fact that CGRP+ terminals forming symmetric or asymmetric synapses were partly segregated in their postsynaptic targets (somata vs. spines) suggests that these synaptic features represent a genuine ultrastructural difference between two types of CGRP terminals.

These observations raise important questions about possible differences in the postsynaptic mediators of synaptic transmission at these two types of CGRP+ synapses. Given our knowledge of the protein composition of thick postsynaptic density (PSD) of asymmetric synapses, one may suggest that the postsynaptic membrane of asymmetric CGRP+ axo‐dendritic and axo‐spinous synapses is endowed with large aggregates of various glutamate receptor subtypes (NMDARs, AMPARs, mGluRs), scaffolding proteins (i.e., PSD‐95), signalling proteins (i.e., CaMKII; Homer), and cytoskeletal components (i.e., actin, calmodulin) (Kennedy 1997, 2000; Ziff 1997; Sheng and Hoogenraad 2007), while the lack of such postsynaptic specialization may presumably be due to a more simplified PSD architecture. It is also possible that transmission at symmetric synapses is largely mediated by CGRP receptors instead of glutamate receptors (see below). Further studies are needed to fully elucidate the composition of the PSDs and determine if such a structural and biochemical difference has an impact upon the signalling properties of these synapses.

### 4.2 Could CGRP Be the Primary Transmitter Released at Some CGRP+ PB‐CeA Synapses?

It is known that CGRP is stored in DCVs (Matteoli et al. 1988; Merighi 2018), and previous EM studies have described CGRP localization around large DCVs in the dorsal horn of the spinal cord in monkeys and humans and in frog nerve muscles (Carlton et al. 1987; Harmann et al. 1988; Matteoli et al. 1988; Merighi 2002). Our immunogold data depict a similar pattern of CGRP immunoreactivity in PB‐CeA terminals in both mice and monkey tissue. While it can be said that most CGRP+ terminals examined in this study contained DCVs, the density of such vesicles varied significantly between terminals, such that some terminals were packed with electron‐lucent vesicles among which were interspersed a few DCVs, while others were enriched in DCVs with less electron‐lucent vesicles. Although this may suggest that PB terminals are heterogeneous in their relative content in CGRP‐containing vesicles, vesicle counts from single ultrathin sections must be interpreted with caution as they do not provide a complete view of the terminals. Future 3D‐EM reconstruction of individual CGRP+ terminals may help address this issue. However, it is difficult to determine whether the number of DCVs is correlated with increased storage or synaptic release of CGRP. The fact that we did not find specific aggregation of CGRP+ DCVs in the presynaptic active zones of either symmetric or asymmetric synapses suggests that CGRP may not have been synaptically released at the time animals used in our study were euthanized. Additional studies that directly examine the experimental conditions under which CGRP‐mediated synaptic transmission occurs at PB‐CeA synapses are needed to further characterize the functional role of CGRP vs. glutamate release at these synapses.

A critical regulator of CGRP‐mediated effects in the CeA is the localization and relative abundance of CGRP receptors in relation to synapses formed by PB‐CeA CGRP+ terminals. There is evidence that CGRP receptor 1 is strongly expressed in the CeA (Kruger et al. 1988; van Rossum et al. 1997) and that CGRP receptor 1 activation in the CeA regulates PKA‐ and NMDA‐mediated synaptic plasticity and pain‐related behaviors (Han et al. 2005). Given recent evidence that CGRP itself acts as a peptidergic modulator of threat behavior in the parabrachio‐amygdaloid pathway (Kim et al. 2024), there may be a subpopulation of PB‐CGRP+/vGluT2‐ terminals that mediate some aspects of pain plasticity and pain‐related behaviors selectively through peptidergic transmission.

### 4.3 Interaction Between CGRP and GluD1 in the CeA

Though GluD1 exhibits strong mRNA and protein expression in the CeA (Konno et al. 2014; Hepp et al. 2015), there is limited knowledge of its subcellular and subsynaptic localization and relationships with specific afferents in this region. In a recent study, Gandhi et al. have shown that GluD1 localizes postsynaptically to CGRP terminals in the soma of mouse CeA neurons (Gandhi et al. 2021). Our confocal microscopy data confirm these observations and extend those to the primate CeA. In line with these findings, our electron microscopic observations revealed strong GluD1‐CGRP colocalization at axo‐somatic synapses in both mice and monkey CeA. In addition, we found that most GluD1 immunogold labeling was localized to the main body, or less frequently, at the edges of synapses formed by CGRP+ terminals, further strengthening the foundation that GluD1 is involved in the regulation of CGRP‐CeA synapses (Gandhi et al. 2021).

Interestingly, both symmetric and asymmetric synapses formed by CGRP+ terminals expressed GluD1. However, the pattern of subsynaptic GluD1 expression at the two subtypes of CGRP+ synapses differed; while GluD1 was heavily expressed in the core of symmetric synapses, it was predominantly found at the edges (i.e., perisynaptic) of asymmetric axo‐dendritic and axo‐spinous synapses. Given that the pre‐embedding immunogold method was used to label GluD1, we cannot rule out the possibility that the lack of GluD1 labeling in the core of some asymmetric synapses may be due to problems for the GluD1 or the secondary gold‐conjugated antibodies to reach their epitopes being hidden within the dense meshwork of protein aggregates in the PSD. This concern has been raised before to explain problems in localizing AMPA and NMDA receptor subunits in the core of asymmetric glutamatergic synapses (Galvan et al. 2006). Although this limitation may explain the lack of synaptic GluD1 expression at asymmetric CGRP+ synapses in the CeA, it is noteworthy that perisynaptic aggregation of group I metabotropic glutamate receptors (mGluRs), a subtype of receptors known to functionally interact with GluD1, is also expressed perisynaptically throughout the CNS (Baude et al. 1993; Hanson and Smith 1999; Galvan et al. 2006; Mansouri et al. 2015; Choi et al. 2026). The possibility for GluD1/group I mGluRs interactions at PB‐CeA CGRP+ synapses, as shown in other brain regions (Suryavanshi et al. 2016; Choi et al. 2026), should be examined.

Although GluD1 has been shown to localize postsynaptically in the core of symmetric synapses in other brain regions, these synaptic contacts involved presynaptic GABAergic terminals (Fossati et al. 2019; Gawande et al. 2021; Choi et al. 2025). In the CeA, the CGRP+ terminals likely express vGluT2 and mediate excitatory synaptic transmission. Consistent with these observations, studies have shown that disruption of GluD1 in the CeA leads to a reduction in mEPSC frequency and amplitude but no changes in mIPSCs (Gandhi et al. 2021). Given that there is reduced GluD1 expression in both inflammatory and neuropathic pain models (Gandhi et al. 2021), it would be prudent to determine how these pain states affect GluD1 expression as well as its localization with CGRP at the ultrastructural level. Studies exploring morphometric measurements associated with changes in strength and physiological properties of CGRP+/GluD1 + synapses in different pain states would further contribute to our understanding of GluD1's role in the morpho‐functional regulation of the synaptic signalling substrate of pain processing in the CeA.

### 4.4 Concluding Remarks

Synaptic transmission and plasticity at PB‐CeA synapses have been well studied in rodents. Date presented in this study further extend this knowledge by deepening our understanding of the anatomical substrate through which nociceptive signals are transmitted through these synapses in both mice and monkeys. Our study provides the first detailed analysis of the ultrastructural features and synaptic organization of CGRP+ PB‐CeA terminals in the primate CeA. The rigorous species comparison of ultrastructural data collected in this study allows us to conclude that the underlying synaptic network that mediates transmission of nociceptive signals at PB‐CeA synapses is highly comparable between primates and rodents, increasing the translational value of our findings to the human brain. Another important contribution of our work is to provide further evidence that CGRP+ terminals in CeA are heterogeneous and form either symmetric or asymmetric synapses. Although this has been reported before, limited attention has been devoted to the potential functional significance of this structural difference between these two types of synapses. Even if our findings do not provide evidence that the signalling through these structurally different synapses displays specific properties, they set the stage for future functional experiments aimed at comparing the properties of symmetric vs. asymmetric PB‐CeA synapses. Our data demonstrate that both types of terminals express vGluT2, thereby confirming the glutamatergic nature of the PB‐CeA projection. Finally, our double immuno‐EM experiments demonstrate strong postsynaptic GluD1 expression in the core of symmetric synapses and at the edges of asymmetric synapses formed by CGRP+ terminals in both mice and monkey CeA, extending recent evidence that GluD1 regulates CGRP+PB‐CeA synapses. Altogether, these findings extend knowledge of the anatomical substrates that mediate transmission of nociceptive signals through the parabrachio‐amygdaloid pathway and lay the foundation for future mechanistic experiments to further decipher how GluD1 contributes to the development, regulation, and maintenance of this synaptic network in control state and in response to ascending nociceptive signals.

## Author Contributions

**Diane Choi:** conceptualization, data curation, formal analysis, investigation, methodology, project administration, validation, visualization, writing – original draft, writing – review and editing. **Rosa M. Villalba:** visualization, writing – review and editing. **Karina Dalal:** data curation, formal analysis, methodology, validation, writing – review and editing. **Jean‐Francois Paré:** conceptualization, investigation, methodology, validation, writing – review and editing. **Shashank M. Dravid:** conceptualization, funding acquisition, investigation, supervision, writing – review and editing. **Yoland Smith:** conceptualization, funding acquisition, investigation, project administration, supervision, writing – review and editing.

## Ethics Statement

The use of animals in this study has been approved by the Texas A&M and Emory University IACUC and is in line with the NIH guidelines. AI has not been used to generate or edit text, figures, and tables presented in this study.

## Conflicts of Interest

The authors declare no conflicts of interest.

## Supporting information


**Table S1:** Percentages of different types of GluD1‐labelled synapses formed by CGRP+ terminals on dendrites, somata, and spines of mouse CEA neurons. The upper half of the table shows the raw values collected from the three mice (RM300, RM302, and RM305) used in this analysis. The lower half depicts the mean (+/− SEM) percentages of terminals involved in the four types of synaptic (synaptic symmetric, synaptic asymmetric, perisynaptic symmetric, and perisynaptic asymmetric) with each postsynaptic target.
**Table S2:** Percentages of different types of GluD1‐labelled synapses formed by CGRP+ terminals on dendrites, somata, and spines of monkey CEA neurons. The upper half of the table shows the raw values collected from the three mice (MR368, MR371, and MR372) used in this analysis. The lower half depicts the mean (+/− SEM) percentages of terminals involved in the four types of synaptic arrangement (synaptic symmetric, synaptic asymmetric, perisynaptic symmetric, and perisynaptic asymmetric) with each postsynaptic target.

## Data Availability

There are no additional data to share publicly from this paper. Should investigators need access to specific data, requests can be submitted to the corresponding author.
