# Supplementary material for: Calcitonin Gene‐Related Peptide (CGRP)‐Containing Terminals in the Central Amygdala of Mice and Monkeys: Ultrastructural Analysis and Subsynaptic Expression of GluD1
Source: Eur J Neurosci. 2026 Jul 16;64(2):e70607. doi: 10.1111/ejn.70607 (PMC13373983; doi:10.1111/ejn.70607)
Supplement: Supplementary file 1 — Table S1: Percentages of different types of GluD1‐labelled synapses formed by CGRP+ terminals on dendrites, somata, and spines of mouse CEA neurons. The upper half of the table shows the raw values collected from the three mice (RM300, RM302, and RM305) used in this analysis. The lower half depicts the mean (+/− SEM) percentages of terminals involved in the four types of synaptic (synaptic symmetric, synaptic asymmetric, perisynaptic symmetric, and perisynaptic asymmetric) with each postsynaptic target. Table S2: Percentages of different types of GluD1‐labelled synapses formed by CGRP+ terminals on dendrites, somata, and spines of monkey CEA neurons. The upper half of the table shows the raw values collected from the three mice (MR368, MR371, and MR372) used in this analysis. The lower half depicts the mean (+/− SEM) percentages of terminals involved in the four types of synaptic arrangement (synaptic symmetric, synaptic asymmetric, perisynaptic symmetric, and perisynaptic asymmetric) with each postsynaptic target. [file EJN-64-0-s001.docx]

**SUPPLEMENTARY TABLES**

**Table 1:** Percentages of different types of GluD1-labeled synapses formed by CGRP+ terminals on dendrites, somata and spines of mouse CEA neurons. The upper half of the table shows the raw values collected from the three mice (RM300, RM302, RM305) used in this analysis. The lower half depicts the mean (+/- SEM) percentages of terminals involved in the 4 types of synaptic arrangement (synaptic symmetric, synaptic asymmetric, peri-synaptic symmetric, peri-synaptic asymmetric) with each post-synaptic target.

|  | **Dendrites (n=56)** | | | | **Soma (n=34)** | | | | **Spines (n=8)** | | | |
| --- | --- | --- | --- | --- | --- | --- | --- | --- | --- | --- | --- | --- |
| **Mice** | Syn-S | Syn-A | Psyn-S | Psyn-A | Syn-S | Syn-A | Psyn-S | Psyn-A | Syn-S | Syn-A | Psyn-S | Psyn-A |
| **RM300** | 78.6% | 0% | 14.3% | 7.1% | 93.3% | 0% | 0% | 6.7% | 100% | 0% | 0% | 0% |
| **RM302** | 65% | 0% | 0% | 0% | 100% | 0% | 0% | 0% | 0% | 0% | 0% | 100% |
| **RM305** | 63.6% | 2% | 4.8% | 31.8% | 95.2% | 2.6% | 0% | 2.2% | 33.3% | 0% | 0% | 66.7% |

|  | **Synaptic** | | **Peri-synaptic** | |
| --- | --- | --- | --- | --- |
|  | *Symmetric* | *Asymmetric* | *Symmetric* | *Asymmetric* |
| **Dendrite** (n=56) | 69.1 ± 4.8% | 3.2 ± 1.6% | 4.8 ± 4.8% | 23.0 ± 7.9% |
| **Soma** (n=34) | 95.2 ± 2.4% | 2.6 ± 2.6% | - | 2.2 ± 2.2% |
| **Spine** (n=8) | 33.3 ± 33.3% | - | - | 66.7 ± 33.3% |

**Table 2:** Percentages of different types of GluD1-labeled synapses formed by CGRP+ terminals on dendrites, somata and spines of monkey CEA neurons. The upper half of the table shows the raw values collected from the three mice (MR368, MR371, MR372) used in this analysis. The lower half depicts the mean (+/- SEM) percentages of terminals involved in the 4 types of synaptic arrangement (synaptic symmetric, synaptic asymmetric, peri-synaptic symmetric, peri-synaptic asymmetric) with each post-synaptic target.

|  | **Synaptic** | | **Peri-synaptic** | |
| --- | --- | --- | --- | --- |
|  | *Symmetric* | *Asymmetric* | *Symmetric* | *Asymmetric* |
| **Dendrite** (n=63) | 74.3 ± 3.0% | 2.9 ± 2.9% | 4.7 ± 2.5% | 18.2 ± 6.9% |
| **Soma** (n=17) | 100.0 ± 0.0% | - | - | - |
| **Spine** (n=6) | - | - | - | 100% |

|  | **Dendrites (n=63)** | | | | **Soma (n=17)** | | | | **Spines (n=8)** | | | |
| --- | --- | --- | --- | --- | --- | --- | --- | --- | --- | --- | --- | --- |
| **Monk.** | Syn-S | Syn-A | Psyn-S | Psyn-A | Syn-S | Syn-A | Psyn-S | Psyn-A | Syn-S | Syn-A | Psyn-S | Psyn-A |
| **MR368** | 68.4% | 0% | 5.3% | 26.3% | 100% | 0% | 0% | 0% | 100% | 0% | 0% | 0% |
| **MR371** | 78.3% | 8.7% | 8.7% | 4.4% | 100% | 0% | 0% | 0% | 80% | 0% | 0% | 20% |
| **MR372** | 76.1% | 0% | 0% | 23.8% | 100% | 0% | 0% | 0% | 0% | 0% | 0% | 0% |
